# Israeli soldiers' intentions and actions toward seeking mental health help: Barriers and facilitators

**DOI:** 10.1002/jclp.23431

**Published:** 2022-08-21

**Authors:** Maayan Schneider, Shirli Werner, Nirit Yavnai, Ariel Ben Yehuda, Leah Shelef

**Affiliations:** ^1^ Paul Baerwald School of Social Work and Social Welfare The Hebrew University of Jerusalem Jerusalem Israel; ^2^ Department of Health and Well‐Being, IDF's Medical Corps Israel Defense Forces Ramat Gan Israel; ^3^ Department of Military Medicine, Faculty of Medicine The Hebrew University of Jerusalem Jerusalem Israel

**Keywords:** barriers and facilitators for seeking help, consulting an MHO, Health Belief Model, help seeking, Help‐Seeking Model, intention to seek help, military service

## Abstract

**Background:**

While military settings may increase psychological distress, soldiers frequently avoid seeking professional help. This study aimed to examine barriers and facilitators associated with intentions to seek help and actually seeking help from a mental health officer (MHO) and how these differ among soldiers who had sought help in the past and those who had not.

**Method:**

This cross‐sectional study included 263 combat and noncombat soldiers. The Health Belief Model and the Help‐Seeking Model were the theoretical framework used to map the potential variables associated with soldiers' decision to seek help.

**Results:**

Stigma and administrative barriers were found to be significant barriers to both the intention to seek help and actually consulting an MHO. These findings were more definitive among combat soldiers. The belief in the effectiveness of mental health treatment was positively associated with the intention to seek help. Positive associations were found between well‐being, perceived seriousness of one's condition, and belief in the effectiveness of mental health care and intention to seek MHO help. Distress and self‐concealment were positively associated with actual consultation with an MHO. Public stigma about seeking help was associated with both the intention to seek mental health assistance and actually consulting an MHO.

**Conclusion:**

Military commanders should make an effort to make soldiers feel safe to seek mental health assistance by creating a supportive organizational atmosphere to reduce the stigma associated with mental health care.

## 1 INTRODUCTION

Military environments are typically highly stressful and liable to increase the risk of mental illness (Castro & Kintzle, 2014). However, despite ongoing efforts of the military to provide professional assistance, many soldiers continue to avoid seeking help (Applegarth et al., 2019; Vogt et al., 2014). Moreover, the rate of mental health consultations in the military is lower than in the general population (Hom et al., 2017), given the reluctance to demonstrate weakness or vulnerability within a military system (Bryan et al., 2012; Castro & Kintzle, 2014; Stecker et al., 2007).

The Israeli Defense Forces (IDF) are a unique setting for examining the matter of seeking mental health treatment. Given that service in the IDF is mandatory, it is a socialization milestone in Jewish Israelis' transition to adulthood (Girsh, 2019; Mayseless & Scharf, 2003). Thus, young Israeli soldiers may avoid seeking help for fear this could lower their medical profile and interfere with their military future (e.g., combat jobs, becoming officers, and proceeding to career service). One critical risk of untreated mental illness is suicide (Bryan et al., 2012; Nock et al., 2014). Readily available psychological support is therefore crucial in any program designed to prevent suicide (Zalsman et al., 2016).

The current study aimed to examine the variables associated with intention to seek help from a mental health officer (MHO) and how these differ among soldiers who actually consulted an MHO. To the best of the authors' knowledge, this is the first study investigating the role of barriers and facilitators involved in seeking mental health treatment in the IDF.

Help seeking is the act of contacting another person for information, advice, treatment, and support when struggling with a particular problem. It is possible to seek help informally from friends and family or formally from qualified sources such as community mental health services (Rickwood et al., 2005). Studies suggest that young people are more likely to seek informal help (Michelmore & Hindley, 2012; Rickwood et al., 2005). However, formal—rather than informal—help has been found to protect against various mental health risks (e.g., Rickwood et al., 2005). Hence, help seeking is essential in stressful circumstances that increase the likelihood of mental illness (Henderson et al., 2013; Stergiopoulos et al., 2020), as untreated mental illness may have grave consequences, such as suicide (Nock et al., 2014). Unfortunately, studies show that many young adults who struggle with mental health difficulties do not seek professional help (Gulliver et al., 2010; Henderson et al., 2013).

The present study focuses on help seeking within the Israeli military. Specifically, it examines soldiers' intention to seek mental health help and actually seeking help. Intentions to seek help are associated with positive attitudes toward seeking professional mental health treatment (Seyfi et al., 2013). Such positive attitudes may result from a positive previous experience, a favorable opinion about help seeking, confidence in receiving treatment, having a good relationship with the care provider, and being familiar with mental health services (Clark et al., 2018).

The realization of help‐seeking intentions (i.e., accepting mental help) is termed “actual help‐seeking behavior” (Seyfi et al., 2013). Actual help seeking is therefore dependent upon individual intentions to seek mental help (Vogel & Wei, 2005). Various variables may act as facilitators or barriers to seeking help. It is necessary to identify these variables to be able to assess at‐risk populations, specifically in the military (Elnitsky et al., 2013; LeFeber & Solorzano, 2019).

Intention to seek help and actual help seeking have become part of many human social behavior theories (Seyfi et al., 2013). The present study leans on the Health Belief Model (HBM) and the Help Seeking Model (HSM) to acquire a full picture of the facilitators and barriers associated with soldiers' intentions and actual help seeking. Facilitating factors are components that increase the likelihood of seeking help in times of need (Clark et al., 2018), while barriers hinder it. The HBM (Rosenstock, 1974) lists six factors that can act as facilitators or barriers to help seeking: perceived susceptibility, perceived seriousness of one's condition, belief in the effectiveness of mental health care, administrative barriers, psychological barriers and self‐efficacy (Cramer, 1999). The HSM (Rickwood et al., 2005) adds self‐concealment and social support. We further expand on each of these variables below.

*Perceived Susceptibility* is the subjective sense of risk of experiencing a mental health condition (Rosenstock, 1974). Some individuals tend to deny any possibility of an eventual mental condition. Others acknowledge that mental illness is possible, albeit consider it improbable. However, a third group exists of individuals who may feel they are at high risk of mental condition (Rosenstock, 1974). Individuals who believe they are susceptible to mental health concerns would seek help more frequently than those who believe they are not at risk (Bird et al., 2020).

*Perceived Seriousness* is the individual perception of the gravity of the condition's impact. An individual may perceive the condition as having medical consequences such as impaired physical or mental functioning for shorter or longer periods or even a risk of death (Rosenstock, 1974). An individual who sees the issue as grave and negatively impacting daily functioning would be more likely to seek help (Bird et al., 2020). An individual seeking help must also consider the perceived benefits and barriers of undergoing treatment (Bird et al., 2020).

*Believing in the effectiveness of treatment*, (i.e., confidence that the treatment will relieve symptoms), will encourage seeking help while skepticism about the effectiveness of mental health care will be a barrier (Stergiopoulos et al., 2020).

Administrative barriers such as treatment costs, not knowing how to access treatment, physical accessibility of treatment, and time constraints hindering treatment may be negatively associated with seeking help. Other psychological barriers are the stigma on mental health disorders or treatment and the concern that help‐seeking might jeopardize one's career development (Stergiopoulos et al., 2020). The present study examines two stigma types: public stigma (i.e., a perception of stigma endorsed by the general population; Vogel et al., 2013), and self‐stigma (i.e., internalization of the public stigma; Watson et al., 2007). Self‐efficacy, the belief in one's own ability to complete or execute a specific task (i.e., believing that one is capable of seeking help; Bandura, 1982), has been associated with help seeking (Lian et al., 2020; Williams & Takaku, 2011). Social support, the number and quality of the relationships one has with others, and their support concept (Pearson, 1986), has also been associated with seeking help (O'Connor, 2013). Self‐concealment is a predisposition to deliberately conceal personal information from others (Larson & Chastain, 1990). People who tend to hide distressing personal information have lesser social support, higher personal stress, and a negative attitude toward seeking professional help.

An additional variable examined in the present study is well‐being, an individual's subjective assessment of mental health and quality of life (Veit & Ware, 1983). The opposite of well‐being is experiencing negative psychological symptoms that reflect distress (Veit & Ware, 1983). While well‐being has been associated with help‐seeking (Laidlaw et al., 2016), distress may lead to despondency raising barriers that risk to prevent seeking professional help (Sanagavarapu & Abraham, 2021).

Several studies have addressed help seeking in the military setting. They have shown that stigma is a major barrier to help seeking in the military, as soldiers voice concern that commanders would treat them differently or that their mates would perceive them as weak (Vogt et al., 2014). A survey conducted by the US Marine Corps revealed a moderate support of help seeking, indicating the stigma attached to mental health problems (Farmer et al., 2015). Administrative barriers and skepticism about the treatment's effectiveness have also emerged as hindering treatment seeking (Hom et al., 2017; Vogt et al., 2014). Support of family and friends (Hom et al., 2017) and perceived self‐efficacy (Keeling et al., 2020) were both positively associated with seeking mental health care. Positive or negative views about help seeking as described above (Clark et al., 2018), revealed differences between combat and noncombat soldiers. Military combat veterans showed a lesser tendency to seek help than noncombat veterans (Ashley & Brown, 2015).

Despite the significance of these studies, none of them have been conducted in the Israeli military. Furthermore, no studies examined barriers and facilitators based on the theoretical framework (Martínez‐Hernáez et al., 2014) of the HBM and the HSM in military settings. Finally, to the best of our knowledge, this is the first study to compare help seeking patterns in combat and noncombat soldiers.

Based on the literature reviewed above, we hypothesized that:

(1) Associations will emerge between barriers and facilitators and the intention to seek help. Specifically,

(a) A negative association will be found between well‐being, administrative barriers, stigma (perceived public stigma and self‐stigma), and self‐concealment, and the intention to seek help.

(b) Positive associations will be found between distress, self‐efficacy, belief in the effectiveness of mental health care, social support, perceived susceptibility, and perceived seriousness of one's condition and the intention to seek help.

(2) A positive association will be found between the intention to seek help and actually consulting an MHO.

(3) Differences will be found in the barriers and facilitators between soldiers who consulted an MHO and those who did not.

Given that this was the first study to examine differences between combat and noncombat soldiers, no specific hypothesis was made about this matter.

## 2 METHOD

### 2.1 Participants

The study participants were 263 soldiers (77.9% males), mean age 19.68 (SD = 1.53), 91.6% Israeli born. Of the participants, 86.7% completed 12 years of school, 52.9% were combat soldiers with a mean military service period of 12.92 months (SD = 5.89). Motivation for military service was high to very high in 63.5% of participants. 68.3% reported they had not considered contacting an MHO during their military service, and 16.7% reported having set a consultation meeting with an MHO over their military service.

### 2.2 Design and settings

The data for this cross‐sectional study were collected between July and September 2019. Research assistants collected the data in two IDF bases, an Air Force base and an infantry base at a meeting set for a day when all the soldiers arrive at the base for training or guidance. The research assistants introduced the study and its purpose and asked those who were willing to participate to sign an informed consent to fill the questionnaire that was distributed to all the base soldiers.

### 2.3 Measurements

All but two of the scales were available and widely used in Hebrew in previous studies. The scales measuring perceived barriers to seeking mental health services and self‐concealment (Elnitsky et al., 2013; Hoge et al., 2004) were translated for the current study (elaborated below).

#### 2.3.1 Background and service‐related variables

Background data included gender, age, and education.

Service‐related variables included months in military service, military profession (combat/noncombat), and motivation to serve in the military. The latter was initially a single item, “How would you define your level of motivation to military service today?” rated on a 5‐point Likert scale ranging from 0 (*very low*) to 4 (*very high*). Since 50% of the participants rated average and below, we recoded the item into two categories (0−2, defined as low‐average, and 3−4, defined as high/very high).

#### 2.3.2 Dependent variables

To measure *Intention to seek MHO help* we used the *General Help Seeking Questionnaire* (Wilson et al., 2005). This measure poses the question “If you were feeling mentally distressed, what is the likelihood that you would ask for professional help from…,” followed by a list of 10 formal and informal supports (Wilson et al., 2005), three of which were adapted to the IDF. Items were rated on a 7‐point Likert scale ranging from 1 (*not likely at all*) to 7 (*most likely*). The Cronbach alpha was *α* = 0.70 in both the previous (Wilson et al., 2005) and present, studies.

A single item measured *Consulting an MHO*, “Have you seen an MHO during your military service?” (*yes/no*).

#### 2.3.3 Independent variables

To measure *well‐being and distress* we used the *Mental Health Inventory* (MHI; Veit & Ware, 1983). This scale includes 38 items rated on a 6‐point Likert scale ranging from 1 (*never*) to 6 (*every day*). The scale is comprised of two subscales: distress (24 items; e.g., “During the last month did you feel depressed?”), and mental well‐being (14 items; e.g., “Over the last month, how long have you felt happy or content with your personal life?”). Mean scores were calculated for each subscale, with higher scores indicating increased well‐being or greater distress. In previous studies (Veit & Ware, 1983), Cronbach alpha ranged 0.82−0.96. In the present study Cronbach *α* was 0.85 for distress, and.83 for well‐being.

One item measured *Perceived susceptibility*, “To what extent do you think you may potentially deal with mental distress in your military service”, and one question measured *Perceived seriousness of one's condition*: “If you were in mental distress, to what extent this distress might affect your daily functioning.” Both items were rated on a 5‐point Likert scale ranging from 0 (*very low*) to 4 (*very high*). Since 50% of the participants rated average and below, we recoded the item as low (0−2) versus high (3−4).

We adapted the *Perceived Barriers to Seeking Mental Health Services Scale* (Hoge et al., 2004) to measure three variables: perceived barriers, perceived stigma and belief in the effectiveness of mental health care. We used 12 of the original 13 items and discarded the item “Mental health care costs a lot of money” as the Israeli military provides free care. Below are the variables measured.

*Administrative barriers* were measured using five items, one constructed by the authors and four taken from the *Perceived Barriers to Seeking Mental Health Services Scale* (Hoge et al., 2004), (e.g., “I do not know where to get mental help”). Each item is scored on a 6‐point Likert scale ranging from 0 (*strongly disagree*) to 5 (*strongly agree*). Cronbach's alpha was *α* = 0.79 in the original scale (Elnitsky et al., 2013). In the current study it was *α* = 0.83.

*Perceived public stigma* was measured by two scales. First, six items were taken from the Perceived Barriers to Seeking Mental Health Services Scale (Hoge et al., 2004). The participants had to rate the extent to which they agreed with the possibility that each item would influence their decision to seek MHO help. To this end they used a 6‐point Likert scale ranging from 0 (*strongly disagree*) to 5 (*strongly agree*) (e.g., “My fellow unit personnel will see me as weak”). A mean score was calculated for each participant. The scale was found to be highly reliable (*α* = 0.84) in a previous study (Elnitsky et al., 2013) as well as in the current one (*α* = 0.88).

Next, we used two sub‐scales of the *Self‐Stigma of Mental Illness Scale* (Watson et al., 2007) to assess perceived public stigma. The “awareness of stereotypes” subscale measures an individual's awareness of common mental illness stereotypes by ten statements that begin with “I believe the majority of the public thinks that…,” followed by common stereotypes. The “acceptance of stereotypes” subscale measures the degree to which the participant considers each of the previous 10 stereotypes as accurate. In each item, “mental illness” was replaced by “Mentally distressed soldier.” For example, “I believe that most people think that it is not easy to rely on mentally distressed soldiers.” Each item is rated on a 9‐point Likert scale ranging from 1 (*strongly disagree*) to 9 (*strongly agree*). In the original study, reliability was high: *α* = 0.89 for awareness and *α* = 0.80 for acceptance scales (Corrigan et al., 2006). In the current study, we obtained *α* = 0.87 and *α* = 0.89 respectively.

*Self‐stigma* was measured using the “self‐application of a stereotype” subscale from the *Self‐Stigma of Mental Illness Scale* (Watson et al., 2007), which measures internalization of a stereotype. Since our sample was nonclinical, we replaced “because I have mental distress” with “if I were struggling with mental distress”. Ten statements were rated on a 9‐point Likert scale ranging from 1 (*strongly disagree*) to 9 (*strongly agree*) (Watson et al., 2007). Reliability was *α* = 0.72 in a previous study (Corrigan et al., 2006) and *α* = 0.91 in the current study.

*Belief in the effectiveness of treatment* was measured using two items developed by the authors and two taken from the *Perceived barriers to seeking mental health services* (e.g., “I do not believe that military mental‐health professionals and mental health care work”; Hoge et al., 2004). Items were rated on a 5‐point Likert scale ranging from 1 (*strongly disagree*) to 5 (*strongly agree*), with a current reliability of *α* = 0.73.

*Self‐concealment* was measured using the 10‐item *Self‐Concealment Scale* (SCS; Larson & Chastain, 1990) addressing three topics: (a) Desire to keep things to oneself (e.g., “There are a lot of personal things that I keep to myself.” (b) Attitudes toward a painful secret or negative thoughts about oneself not shared with anyone (e.g., “I have negative thoughts about myself that I never share with people”). (c) Concern about the disclosure of hidden personal information (e.g., “If I share all my secrets with my friends, they will like me less”). Each item was rated on a 5‐point Likert scale ranging from 1 (*strongly disagree*) to 5 (*strongly agree*). Original reliability was *α* = 0.83, and in the current study it was *α* = 0.91.

*Self‐efficacy* was measured using the 10 items of the *General Self‐Efficacy Scale* (GSE; Schwarzer & Jerusalem, 1995). Items were rated on a 4‐point Likert scale ranging from 1 (*not at all true*) to 4 (*always true*) (e.g., “I can always manage to solve difficult problems if I try hard enough”). The scale had good internal reliability (*α* = 0.91) in a previous study (Schwarzer & Jerusalem, 1995) and in the current study as well (*α* = 0.91).

*Social support* was measured via the 12 items of the *Multidimensional Perceived Social Support Scale* (MPSS; Zimet et al., 1988) (e.g., “My family truly tries to help me”). Items are rated on a 7‐point Likert scale, ranging from 1 (*strongly disagree*) to 7 (*strongly agree*). A higher score indicates greater perceived social support. A mean overall social support score was calculated. Internal reliability was high in previous studies (*α* = 0.85−0.91, Zimet et al., 1988) and in the present one (*α* = 0.88).

### 2.4 Statistical analyses

To analyze the data, we used SPSS (version 20.0). Statistical significance was set at *p* = 0.05. Descriptive statistics served to present the sample and the main study variables.

To analyze the differences in consulting an MHO as manifested in background variables and type of military service, we used *χ*² and the Mann–Whitney test (for asymmetric distribution). We also used the Mann–Whitney test to examine differences in the independent variables between combat and noncombat soldiers. Spearman's correlations and the Mann–Whitney test were used to examine the association between independent variables and an intention to seek MHO help, and the association between the intention to seek MHO help and actually consulting an MHO. Finally, to test the fourth hypothesis, we performed a generalized linear model (GLM) that examined which variables were most strongly associated with the intention to seek help and actual consultation with an MHO. Only significant and borderline variables found in the univariate meeting with an MHO (yes/no, Table 4) were inserted into the GLM. Additionally, we tested the model examining actual consultation with and without previous intention to seek MHO help.

## 3 RESULTS

### 3.1 Descriptive statistics

Table 1 presents the descriptive statistics of the study variables of all the study participants. Two variables were rated slightly higher than the midpoint of the scale: intention to seek help, and well‐being. Mean social support and self‐efficacy were high.

**Table 1:** Descriptive statistics of the study variables (*N* = 263).

Variables	*N*	*M*	SD	Min	Max	Range
Dependent variables						
Intention to seek MHO help	258	4.00	2.00	1.0	7.0	1−7
Independent variables						
Mental health						
Distress	260	2.69	0.63	1.0	4.67	1−6
Well‐being	260	3.49	0.72	1.0	5.36	1−6
Perceived susceptibility	263	2.00	1.15	0.0	4.0	0−4
Perceived seriousness	245	2.34	1.22	0.0	4.0	0−4
Administrative barriers	245	1.95	1.30	0.0	5.0	0−5
Public stigma						
Awareness of stereotype	251	3.86	1.50	1.0	9.0	1−9
Agreement to a stereotype	243	2.98	1.46	1.0	8.4	1−9
Public stigma toward seeking help	211	2.28	1.32	0.0	5.0	0−6
Self‐stigma	247	3.03	1.65	1.0	8.6	1−9
Self‐concealment	244	2.43	1.00	0.9	5.0	1−5
Belief in the effectiveness of mental health care	262	2.77	0.57	1.0	5.0	1−5
Self‐efficacy	243	3.30	0.63	1.0	4.00	1−4
Social support	245	5.73	1.36	1.0	7.0	1−7

Abbreviations: MHO, mental health officer; SD, standard deviation.

As described above, administrative barriers were measured using five items. Four barriers were scored less than the midpoint of the scale (perceiving it difficult to schedule an appointment with an MHO [*M* = 2.08 ± 1.77]; getting time off work for treatment [*M* = 1.93 ± 1.75]; not knowing where to get help [*M* = 1.46 ± 1.50]; and not having adequate transportation [*M* = 1.24 ± 1.52]). However, the strongest barrier the participants faced was the perception that mental health care in the military could potentially harm the individual's military score (*M* = 3.02 ± 1.84).

#### 3.1.1 Differences in the intention to seek MHO help and actually consulting an MHO by background and service‐related variables

No gender differences emerged in the intention to seek MHO help (*U* = 5235.0, *p* = 0.508). However, *χ*² tests (Table 3) show that more male soldiers actually consulted an MHO (61.4%) than female soldiers (38.6%). No differences were found in the intention to seek help according to years of education (Kruskal−Wallis [for multiple categories] *χ*² = 3.916, *df* = 2, *p* = 0.141).

In terms of service‐related variables, no association emerged between motivation to serve in the military and intention to seek MHO help (*U* = 7638.5, *p* = 0.903). As shown in Table 2, a higher number of soldiers who reported low to average motivation for service actually consulted an MHO than soldiers who reported high to very high motivation for service (59.1% vs. 40.9%).

**Table 2:** Differences in actually consulting an MHO according to background and service‐related variables (*χ*² Tests)

	Actually consulting an MHO						*χ*²	*p*
	No (*n* = 219; 83.7%)		Yes (*n* = 44; 16.7%)		Total			
	*n*	%	*n*	%	*N*	%		
Gender								
Female	41	18.8	17	38.6	58	22.1		
Male	177	81.2	27	61.4	204	77.9	8.35	0.009
Years of education								
Less than 12 years	3	1.4	1	2.3	4	1.5		
12 years	191	87.2	37	84.1	228	86.7		
Over 12 years	25	11.4	6	13.6	31	11.8	0.39	0.82
Military profession								
Combat	134	61.2	5	11.4	139	52.9		
Noncombat	85	38.8	39	88.6	124	47.1	36.49	<0.001
Motivation to serve in the military								
Low‐average	70	32.0	26	59.1	96	36.5		
High‐very high	149	68.0	18	40.9	167	63.5	11.63	0.001

Abbreviation: MHO, mental health officer.

Table 3 also presents the differences between combat and noncombat soldiers' intentions to seek help in terms of each of the independent variables. As shown, combat soldiers reported weaker intentions to seek help than Noncombat soldiers (*M* = 3.72 ± 2.08 vs. 4.32 ± 1.88; *U* = 6923.0, *p* = 0.022). Fewer combat soldiers actually consulted an MHO than noncombat ones (11.4% vs. 88.6%) (Table 2). As for the independent variables, compared to noncombat soldiers, combat soldiers faced more administrative barriers and greater public stigma. Combat soldiers also reported a higher level of self‐stigma, self‐efficacy and social support compared to noncombat soldiers (Table 3).

**Table 3:** Differences between combat and noncombat soldiers

Variables	Noncombat			Combat			*z*	*U*	*p*
	*n*	Mean	SD	*n*	Mean	SD			
Dependent variables Intent to seek help	122	4.32	1.88	138	3.72	2.08		6923.0	0.022
Mental health									
Distress	122	65.79	17.35	138	62.31	11.96	−1.372	7588.00	0.17
Well‐being	122	48.03	10.64	138	48.57	9.56	−0.240	8273.00	0.81
Perceived susceptibility	124	2.09	1.23	139	1.91	1.07	−1.074	7977.50	0.28
Perceived seriousness	117	2.26	1.26	128	2.42	1.18	−1.100	6901.00	0.27
Administrative barriers	100	1.50	1.14	135	2.32	1.31	−4.90	4727.50	<0.001
Public stigma									
Awareness of stereotype	115	3.79	1.60	136	3.92	1.42	−0.792	7366.00	0.42
Agreement to a stereotype	113	2.84	1.53	143	3.10	1.39	−1.789	6368.00	0.07
Public stigma seek help	77	1.72	1.16	134	2.60	1.30	−4.67	3163.50	<0.001
Self‐stigma	113	2.76	1.67	134	3.26	1.61	−2.817	5996.50	0.005
Self‐concealment	109	2.48	1.02	135	2.40	0.99	−0.654	6999.00	0.51
Belief in the effectiveness of mental health care	124	2.78	0.59	138	2.77	0.56	−0.420	8302.00	0.67
Self‐efficacy	106	3.13	0.74	137	3.44	0.48	−3.321	5460.50	0.001
Social support	108	5.45	1.60	137	5.96	1.09	−2.048	6277.00	0.04

*Note*: Mann−Whitney (asymmetric distribution).

Abbreviation: SD, standard deviation.

#### 3.1.2 Association between independent and dependent variables

Spearman's correlations between all the independent variables and the dependent variable of intention to seek MHO help revealed four significant associations: well‐being (*r* = 0.16; *p* = 0.05), administrative barriers (*r* = −0.14; *p* = 0.05), public stigma on seeking help (*r* = −0.24; *p* = 0.001), and belief in the effectiveness of mental health care (*r* = 0.13; *p* = 0.05).

Table 4 presents independent variable differences between soldiers who actually consulted an MHO and those who did not. Soldiers who consulted an MHO reported higher mental distress, higher perceived seriousness of their problem, higher intention to seek help and a higher level of self‐concealment than soldiers who did not consult an MHO. Soldiers who consulted an MHO reported fewer administrative barriers, lower levels of perceived public stigma about mental health care, lower levels of self‐efficacy and less social support than soldiers who did not consult an MHO.

**Table 4:** . Differences in independent variables between soldiers who actually consulted an MHO

Variables	Actually consulting an MHO											
	No			Yes			Total			*z*	U	*p*
	*n*	Mean	SD	*n*	Mean	SD	*N*	Mean	SD			
Mental health												
Distress	217	2.62	0.58	43	3.04	0.75	260	2.69	0.63	−3.25	3200.50	0.001
Well‐being	217	3.53	0.69	43	3.29	0.83	260	3.49	0.72	−1.88	3816.50	0.059
Perceived susceptibility	219	1.95	1.11	44	2.23	1.32	263	2.00	1.15	−1.32	4226.50	0.185
Perceived seriousness	202	2.25	1.17	43	2.77	1.34	245	2.34	1.22	−2.88	3171.00	0.004
Administrative barriers	206	2.02	1.32	39	1.56	1.10	245	1.95	1.30	−2.00	3206.00	0.045
Public Stigma												
Awareness of stereotype	210	3.82	1.51	41	4.06	1.47	251	3.86	1.50	−1.08	3846.00	0.280
Agreement to a stereotype	208	3.03	1.63	39	3.06	1.80	247	3.03	1.65	−0.11	4009.00	0.900
Public stigma toward seeking help	182	2.37	1.30	29	1.75	1.30	211	2.28	1.32	−2.38	1913.00	0.017
Self‐stigma	204	2.99	1.46	39	2.93	1.50	243	2.98	1.46	−0.29	3858.00	0.765
Self‐concealment	206	2.34	0.96	38	2.94	1.10	244	2.43	1.00	−3.08	2680.50	0.002
Belief in the effectiveness of mental health care	218	2.74	0.56	44	2.96	0.59	262	2.77	0.57	−1.89	3938.50	0.058
Self‐efficacy	205	3.39	0.56	38	2.84	0.78	243	3.30	0.63	−4.04	2288.50	<0.001
Social support	207	5.84	1.29	38	5.17	1.60	245	5.74	1.36	−2.39	2978.50	0.017
Intent to seek help	215	3.87	1.978	43	4.65	2.05	258	4.00	2.00	−2.38	3571.00	0.017

*Note*: Mann−Whitney (asymmetric distribution).

Abbreviations: MHO, mental health officer; SD, standard deviation.

#### 3.1.3 Association between the intention to seek MHO help and actually consulting an MHO

A Mann−Whitney test showed that the association between the intention to seek MHO help and actually consulting an MHO was significant (*z* = −2.381; *U* = 3571.00; *p* = 0.017). The mean of intention to seek MHO help was significantly higher among soldiers who consulted an MHO (*M* = 4.65, SD = 2.05) compared to soldiers who did not (*M* = 3.87, SD = 1.98).

#### 3.1.4 Association between the research variables, intention to seek MHO help, and actually consulting an MHO

The variables regarding differences between soldiers who actually consulted an MHO and those who did not (Table 4) that were found significant or borderline significant were inserted into GLMs together with the outcome variables of intention to seek help and actually seeking MHO help. Table 5 displays the results. Positive associations emerged between well‐being, perceived seriousness of one's condition, and belief in the effectiveness of mental health care, and an intention to seek MHO help. Public stigma was negatively associated with the intention to seek MHO help. Table 6 reveals that the distress and self‐concealment variables are positively associated with both the intention to seek MHO help (6a) and no intention to seek MHO help (6b). Public stigma and self‐efficacy were negatively associated with actually consulting an MHO (Table 6).

**Table 5:** Generalized linear model (GLM) to examine intentions to seek mental health help

Independent variables	*B*	Exp (B) (OR)	95% Confidence interval		Sig.
			Lower	Upper	
Intercept	1.397	4.004	0.153	106.905	0.403
Distress	−0.022	0.978	0.541	1.766	0.941
Well‐being	0.518	1.679	1.079	2.613	0.022
Perceived seriousness	0.291	1.338	1.049	1.707	0.019
Administrative barriers	−0.116	0.891	0.696	1.1398	0.355
Public stigma seek help	−0.356	0.701	0.550	0.893	0.004
Self‐concealment	0.116	1.123	0.796	1.584	0.510
Belief in the effectiveness of mental health care	0.500	1.649	1.002	2.715	0.049
Self‐efficacy	−0.253	.777	0.453	1.330	0.357
Social support	0.061	1.063	0.819	1.379	0.646

*Note*: GLM; Final model significant *p* = 0.001

**Table 6:** Generalized linear model (GLM) to examine actually meeting with an MHO

Independent variables	*B*	Exp (B) (OR)	95% Confidence interval		Sig.
			Lower	Upper	
6a. Regression including intentions to seek mental health help (*p* < 0.001)					
Intercept	0.170	1.185	0.703	1.998	0.524
Distress	0.100	1.105	1.006	1.214	0.038
Well‐being	0.035	1.035	0.964	1.112	0.342
Perceived seriousness	0.0024	1.025	0.985	1.066	0.223
Administrative barriers	−0.029	0.971	0.934	1.010	0.143
Public stigma seek help	−0.051	0.950	0.913	0.988	0.011
Self‐concealment	0.065	1.067	1.010	1.127	0.021
Belief in the effectiveness of mental health care	−0.005	0.995	0.919	1.078	0.906
Self‐efficacy	−0.119	0.887	0.814	0.967	0.006
Social support	−0.017	0.983	0.943	1.025	0.424
Intentions to seek mental health help	0.014	1.014	0.992	1.037	0.218
6b. Regression without intentions to seek mental health help (*p* < 0.001)					
Intercept	0.199	1.220	0.721	2.064	0.458
Distress	0.105	1.111	1.010	1.221	0.030
Well‐being	0.037	1.037	0.967	1.113	0.309
Perceived seriousness	0.031	1.031	0.992	1.072	0.123
Administrative barriers	−0.033	0.968	0.930	1.007	0.102
Public stigma seek help	−0.058	0.944	0.908	0.981	0.004
Self‐concealment	0.068	1.070	1.013	1.131	0.016
Belief in the effectiveness of mental health care	−0.002	0.998	0.921	1.081	0.964
Self‐efficacy	−0.131	0.877	0.805	0.956	0.003
Social support	−0.010	0.990	0.950	1.031	0.627

*Note*: GLM; Final model 5a and 5b significant *p* < 0.001

Abbreviation: MHO, mental health officer.

## 4 DISCUSSION

The present study examined the association between soldiers' intentions to seek mental health help and actually consulting an MHO and related barriers and facilitators. In line with hypothesis one and previous studies conducted in armed forces around the world (Applegarth et al., 2019; Elnitsky et al., 2013), findings showed that public stigma toward seeking help was negatively associated with both the intention to seek mental health assistance and actually consulting an MHO. Furthermore, in line with earlier studies (Applegarth et al., 2019; Hoge et al., 2004), combat soldiers reported higher levels of public‐ and self‐stigma compared to noncombat soldiers. In addition, combat soldiers reported lower intention to seek help and were less likely to actually seek help compared to noncombat soldiers. Put together, these findings may be explained by the concern of combat soldiers that mental health care will have negative implications for their functioning in the military. Indeed, previous studies highlight the concern that the stigma attached to seeking help would make commanders and peers perceive help‐seeking as an excuse to evade certain unwelcome military chores or as being weak and unfit to be in the team unit (Bryan et al., 2012; Hom et al., 2017; LeFeber & Solorzano, 2019; Zinzow et al., 2013).

In our study, soldiers who actually consulted an MHO reported higher mental distress, higher perceived seriousness of their problem, higher intention to seek help and a higher level of self‐concealment than soldiers who did not consult an MHO. As expected, the findings of this study supported the notion that the perceived seriousness of one's condition and the belief that mental health care is effective are both positively associated with the intention to seek help. These findings are consistent with studies conducted among military personnel, which found that lack of confidence in the effectiveness of psychiatric treatment was one of the barriers to seeking help (Sayer et al., 2009), whereas soldiers who believed in mental health care tended to seek psychological help (Adler et al., 2015).

Combat soldiers reported facing greater administrative barriers than noncombat ones. However, accessibility to an MHO does not appear to be the main administrative barrier, given that in the IDF MHOs are integrated into the military brigade‐level, especially to reduce the help‐seeking stigma for combat soldiers, it enables soldiers to consult with an MHO when needed (Shelef et al., 2015). Rather, and in line with previous studies (Elnitsky et al., 2013; Hoge et al., 2004), the main administrative barrier voiced by combat soldiers in this study was the concern that seeking help may have negative implications for their military career (Shelef et al., 2015). This unfortunate finding shows that despite efforts made by the military to disassociate between psychological help and negative implications to military roles (Castro & Kintzle, 2014; Wolfe‐Clark & Bryan, 2017), soldiers are still worried about negative consequences. Military leaders should continue making efforts to remove this barrier and reduce the stigma of seeking help during the military service.

An additional explanation for the help‐seeking difference between combat and noncombat soldiers may be related to prestudy differences between these groups. Specifically, during the enlistment process all soldiers undergo screening that includes intellectual evaluation (parallel to IQ), evaluation of capability to adapt to military service circumstances (e.g., transition from civilian life to a military environment, military profession, dealing with the peer group, etc.), and evaluation of the motivation to serve in combat units. This three‐part screening helps determine the recruit's quality category and whether he will be assigned combat or noncombat duties. Naturally, these scores are also indicated e differences between soldiers' personal, medical, and mental resources (Shelef et al., 2015). Thus, noncombat soldiers, some of whom were categorized as such due to their mental profile or adaptation difficulties, intended more often to seek help and were more likely to actually consult an MHO.

The Theory of Planned Behavior (Ajzen, 1991), which maintains that the best predictor of behavior is the intention to exhibit that behavior, support our findings that intention to seek MHO help is positively associated with actually consulting an MHO. Similarly, the findings are also in line with the sociobehavioral model (Andersen, 1995) that asserts that intentions to seek help predict help‐seeking behaviors. Nevertheless, similarly to other studies on help‐seeking (Werner et al., 2019) our study also showed that help‐seeking is not solely related to intentions, but rather other variables are also of great importance in understanding help‐seeking.

In line with the above, several variables in our study were found to have differential relationships with intentions and with actual help‐seeking. Specifically, contrary to our hypothesis, self‐concealment was positively associated only with actually consulting an MHO, but not with an intention to seek MHO help. Further, a positive association was found between well‐being and intentions to seek help; while those who actually consulted an MHO reported lower well‐being. Additionally, soldiers who reported weaker social support and lower self‐efficacy were more likely to consult an MHO. One possible explanation for the differing findings regarding intentions and actual help seeking may have to do with distress levels. That is, when in distress soldiers make the effort to use the help that they know is needed for them. In other words, when faced with distress soldiers must overcome the barriers that prevent their intention to seek help. The findings from one study among US military veterans found, for example, that different distress components may lead to different behaviors regarding the intention to seek help or consult an MHO (Blais et al., 2014). Dysphoria severity was found to uniquely and positively correlate with intention to seek mental health care; higher avoidance severity predicted less treatment seeking, while higher reexperiencing severity predicted more treatment seeking (Blais et al., 2014).

Finally, regarding gender differences, the finding in the current study that more females compared to male soldiers actually consulted an MHO may be explained by the greater inclination of female veterans to use help in general (Duncan et al., 2020) as well as by the fact that none of the combat soldiers were female.

## 5 LIMITATIONS

One limitation of this study is its cross‐sectional design where the results do not reflect causality of change over time, and actual seeking help was measured retrospectively. Second, the research population consisted of a convenience sample taken from two specific military bases. Third, self‐report data are susceptible to bias and social desirability. Fourth, our research population was nonclinical and most of the participants did not experience psychological distress. Lastly, while the HBM and HSM theories provided a broad framework for this study, they may have not covered all the factors involved in the process of seeking help in the military.

Future studies are warranted to investigate factors concerning military stress characteristics that the present study did not investigate, and their relationships with and contribution to existing theories and the soldiers' attitudes toward psychiatric treatment. Future research should also study a more diversified military population in terms of military service and actually consulting an MHO.

### 5.1 Conclusion and implications

Findings from this study highlighted the importance of measuring various barriers and facilitators of help‐seeking, as suggested by the HBM and HSM theories given that the various variables have a differential impact on help‐seeking by soldiers. Further, the findings of this study have a significant theoretical contribution highlighting the conceptual difference between intentions and actual consultation with an MHO. Specifically, this means that faced with significant distress, soldiers may make actual use of the offered services even if they had not intended to previously.

Regarding the HBM theoretical model, the present study's findings revealed an association between the components of perceived seriousness, administrative barriers, public stigma toward seeking help, self‐efficacy, and actually consulting an MHO. Regarding the HSM theory, self‐concealment and social support were both associated with consulting an MHO. Specifically, perceived seriousness and belief in the effectiveness of mental health care were positively associated with intentions to seek MHO help, and self‐efficacy was negatively associated with actually consulting an MHO.

From a practical perspective, a need arises to help soldiers recognize the importance of seeking mental health assistance when necessary. Military commanders have an extremely important role in creating a supportive organizational atmosphere that helps reduce the stigma associated with seeking mental health care and encouraging soldiers to seek such help.

## CONFLICT OF INTEREST

The authors declare no conflict of interest.

### 1 PEER REVIEW

The peer review history for this article is available at https://publons.com/publon/10.1002/jclp.23431

## ETHICS STATEMENT

The study was approved by the IDF Human Research Review Board and by the Committee of Human Subject Research of the Medical Corps Helsinki Committee (No. 1987‐2019). Participation did not entail any compensation.

## Data Availability

The data that support the findings of this study are available from the corresponding author upon reasonable request.
